# Endoscopic Endonasal Approach Versus Transcranial Microsurgery in the Management of Skull Base Tumors With Optic Chiasm Compression: A Systematic Review and Meta-Analysis

**DOI:** 10.7759/cureus.104297

**Published:** 2026-02-26

**Authors:** Jesús Jiménez-Sánchez, Rashell Danae Fiallos Baldeón, Karim N Zamora-Amezcua, Juan Felipe Buitrago Navarro, Hugo David Miranda De La Paz, Andrés Pérez García, Montserrat Ceja, Santiago Alejandro Chávez Fuenmayor

**Affiliations:** 1 Neurosurgery, Hospital Infantil de México Federico Gómez, Mexico City, MEX; 2 Medicine, Universidad Regional Autónoma de los Andes, Ambato, ECU; 3 Neurosurgery, Hospitales Puerta de Hierro Andares, Zapopan, MEX; 4 Medicine, Universidad Industrial de Santander, Bucaramanga, COL; 5 Medicine, Universidad del Valle de México, Guadalajara, MEX; 6 Medicine, Universidad de las Américas Puebla, Puebla, MEX; 7 General Internal Medicine, Universidad Autónoma de Guadalajara, Guadalajara, MEX; 8 Medicine, Universidad de las Américas, Quito, ECU

**Keywords:** a systematic review, endonasal endoscopic surgery, optic chiasm, skull base tumor resection, transcranial microsurgery, visual outcome

## Abstract

Skull base tumors, which compress the optic chiasm, include meningiomas, craniopharyngiomas, and pituitary macroadenomas and pose a serious neurosurgical problem. The best surgical route, that is, endoscopic endonasal (EEA) versus microsurgical transcranial (MTA), is still subject to debate. This systematic review and meta-analysis compares the clinical outcomes, visual function, and complications between these approaches. This research followed the guidelines of the Preferred Reporting Items for Systematic Reviews and Meta-Analyses (PRISMA) 2020. A comprehensive search of PubMed, EMBASE, and Scopus was performed until May 17, 2025, for comparative studies providing data on EEA vs. MTA for skull base tumors with documented optic apparatus involvement. The primary outcomes were the rate of gross total resection (GTR) and the rate of post-operative visual improvement. Secondary outcomes included the rates of cerebrospinal fluid (CSF) leak, new cranial nerve deficit, endocrine worsening, and other medical complications. Data were combined with a random-effects model, and heterogeneity was evaluated with the use of I² statistics.

Seven studies met the inclusion criteria with a total of 582 patients (EEA: 287; MTA: 295). No significant difference could be found for GTR rates between EEA and MTA (RR = 1.09; 95% CI: 0.95-1.26; p = 0.22). EEA was associated with a significantly greater rate of post-operative visual improvement (RR = 1.32; 95% confidence interval: 1.08-1.61; p = 0.007). There was a significantly higher rate of CSF leak in the EEA cohort (RR = 3.45; 95% CI: 1.68-7.09; p = 0.0007). Rates of new cranial nerve deficits (RR = 0.65; 95% CI: 0.39-1.08; p = 0.10), endocrine worsening (RR = 0.87; 95% CI: 0.64-1.18; p = 0.37) did not differ significantly. For tumors at the skull base that compress the optic chiasm, EEA and MTA are associated with similar rates of gross total resection. EEA was associated with superior rates of visual function restoration, but with a greater risk for CSF leakage. The decision on which approach to take should be made on an individualized basis, weighing the importance of visual recovery versus the risk of developing certain complications.

## Introduction and background

Skull base tumors with mass effect on the optic chiasm and anterior visual pathways, including meningiomas of the tuberculum sellae and planum sphenoidale, craniopharyngiomas, and large pituitary adenomas, are an important subset of neurosurgical pathology in which preservation and restoration of vision is a critical goal [1]. The surgical approach to these lesions has changed considerably over the last few decades and may be divided into two main corridors, the traditional microsurgical transcranial approach (MTA) and the recently and increasingly used endoscopic endonasal approach (EEA) [2,3].

The MTA, using various frontolateral, pterional, or interhemispheric corridors, provides a familiar anatomic perspective, direct visualization of the optic nerves and chiasm, and extensive access to the bimanual microsurgical dissection [4]. Conversely, the EEA allows for a ventral, midline pathway with an opportunity for early devascularization of the tumor base, avoidance of retraction of the brain, and direct access to the medial optic canal without having to manipulate the optic nerves or brain [5,6]. Proponents of EEA cite the possibility of superior visual results as a result of this direct decompression of the inferior and medial aspect of the optic apparatus [7].

Despite the theoretical benefits of each approach, the comparative efficacy and safety profile is still controversial, and high-quality evidence is limited. While there are many single-center series that report good outcomes for both techniques, good quality data comparing the two techniques is limited and often conflicting [8,9]. These studies are often underpowered, methodologically heterogeneous, and subject to significant selection bias, as the choice of approach is non-randomized and influenced by factors such as tumor laterality, vascular encasement, and surgeon experience. Some studies indicate EEA provides improved visual recovery at the expense of a higher rate of cerebrospinal fluid (CSF) leak, but others report equivalent results [10,11]. This debate continues to unfold, driven by the steep learning curve related to EEA, institutional bias, and heterogeneity of tumor types included [12,13].

Therefore, a synthetic analysis of the comparative literature that exists is necessary to inform decision-making in the surgical setting. This systematic review and meta-analysis are designed to compare the two dominant surgical strategies, EEA and MTA, in the management of skull base tumors with optic chiasm compression, in terms of the oncologic outcomes, visual function, and surgical morbidity.

## Review

Methods

This systematic review was carried out in line with the Preferred Reporting Items for Systematic Reviews and Meta-Analyses (PRISMA) 2020 statement [14]. A protocol was developed but not prospectively registered.

Eligibility Criteria

Studies were selected according to the Population, Intervention, Comparison, Outcomes, and Study (PICOS) design methodological framework, which allowed the establishment of structured criteria for the inclusion and exclusion of relevant scientific evidence. Regarding the population, adult patients aged 18 years and older who underwent primary surgical resection for benign skull base tumors, specifically meningiomas, craniopharyngiomas, or pituitary macroadenomas, were included. Additionally, patients were required to have radiographic and/or clinical evidence of optic chiasm compression to ensure clinical homogeneity and the relevance of visual outcome assessment.

With respect to the intervention, only studies evaluating tumor resection performed through a purely endoscopic endonasal approach (EEA) were considered. This technique represents a minimally invasive surgical strategy that provides direct access to midline skull base lesions and may offer advantages in terms of surgical visualization and tissue manipulation. The comparator group included studies evaluating tumor resection performed via a microsurgical transcranial approach (MTA), which encompasses conventional open cranial techniques traditionally used for the management of these lesions.

The primary outcomes of interest were the rate of gross total resection (GTR) and the rate of postoperative visual improvement, as these endpoints are critical indicators of surgical effectiveness and functional recovery. Secondary outcomes included the incidence of postoperative cerebrospinal fluid (CSF) leak, the occurrence of new or worsened cranial nerve deficits other than optic nerve involvement, postoperative endocrine deterioration, and general medical complications. Additional outcomes, such as recurrence-free survival and volumetric extent of resection, were identified as clinically relevant; however, they were not included in the quantitative synthesis due to insufficient and inconsistent reporting across the eligible studies. Instead, these outcomes are summarized narratively in the Discussion section.

Regarding study design, only comparative studies directly evaluating outcomes between EEA and MTA were included. Eligible designs comprised retrospective and prospective cohort studies, as well as case-control studies. Case series, narrative reviews, editorials, and non-comparative investigations were excluded to maintain methodological rigor and ensure the reliability of comparative analyses.

Study Design

Comparative studies (retrospective or prospective cohort studies, case-control studies) directly compare EEA and MTA. Case series, reviews, editorials, and non-comparative studies were excluded.

Source of Information and Search Strategy

A systematic search of the literature was conducted in three electronic search engines (PubMed/MEDLINE, EMBASE, and Scopus). The database search was performed from the inception of each database to May 17, 2025. The search strategy was a combination of Medical Subject Headings (MeSH) terms and free-text keywords related to ("endoscopic endonasal" OR "endonasal") AND ("transcranial" OR "microsurgical" OR "pterional" OR "frontolateral") AND ("optic chiasm" OR "visual outcome" OR "suprasellar") AND ("meningioma" OR "craniopharyngioma" OR "pituitary adenoma"). The complete search strategies for all databases are included in Appendix A. Reference lists of identified reviews and included articles were manually searched for additional eligible studies.

Study Selection and Data Collection Process

All titles and abstracts retrieved from the database search were screened to assess potential eligibility. Full-text articles of studies considered potentially relevant were subsequently reviewed in detail to determine compliance with the predefined inclusion criteria. Any discrepancies identified during the selection process were resolved through discussion and consensus, with senior methodological oversight when necessary.

Data extraction was performed using a standardized, pre-piloted form to ensure consistency and accuracy. The information collected from each included study comprised the first author, year of publication, study design, country of origin, patient demographics (age and sex), tumor pathology, number of patients in the EEA and MTA groups, duration of follow-up, and data corresponding to all pre-specified outcomes.

Risk of Bias Assessment

The methodological quality of the included non-randomized comparative studies was independently evaluated by two reviewers using the Risk of Bias in Non-randomized Studies of Interventions (ROBINS-I) tool [15]. A summary of the domain-level judgments for each study is presented in Appendix B.

Synthesis Methods

Statistical analysis was done with Review Manager (RevMan) software, Version 5.4 (The Cochrane Collaboration, London, England, UK). For dichotomous outcomes, the pooled risk ratio (RR) with 95% confidence interval (CI) was calculated using the Mantel-Haenszel method. A random effects model was used for all meta-analyses. Statistical heterogeneity was measured with the I² statistic. A p-value of < 0.05 was considered statistically significant. For outcomes with zero events in one arm, a continuity correction of 0.5 was applied. Subgroup analysis by tumor pathology (meningioma vs. craniopharyngioma) was planned. Publication bias was to be evaluated by a funnel plot if ≥10 studies were included.

Results

Study Selection

**Figure 1:**
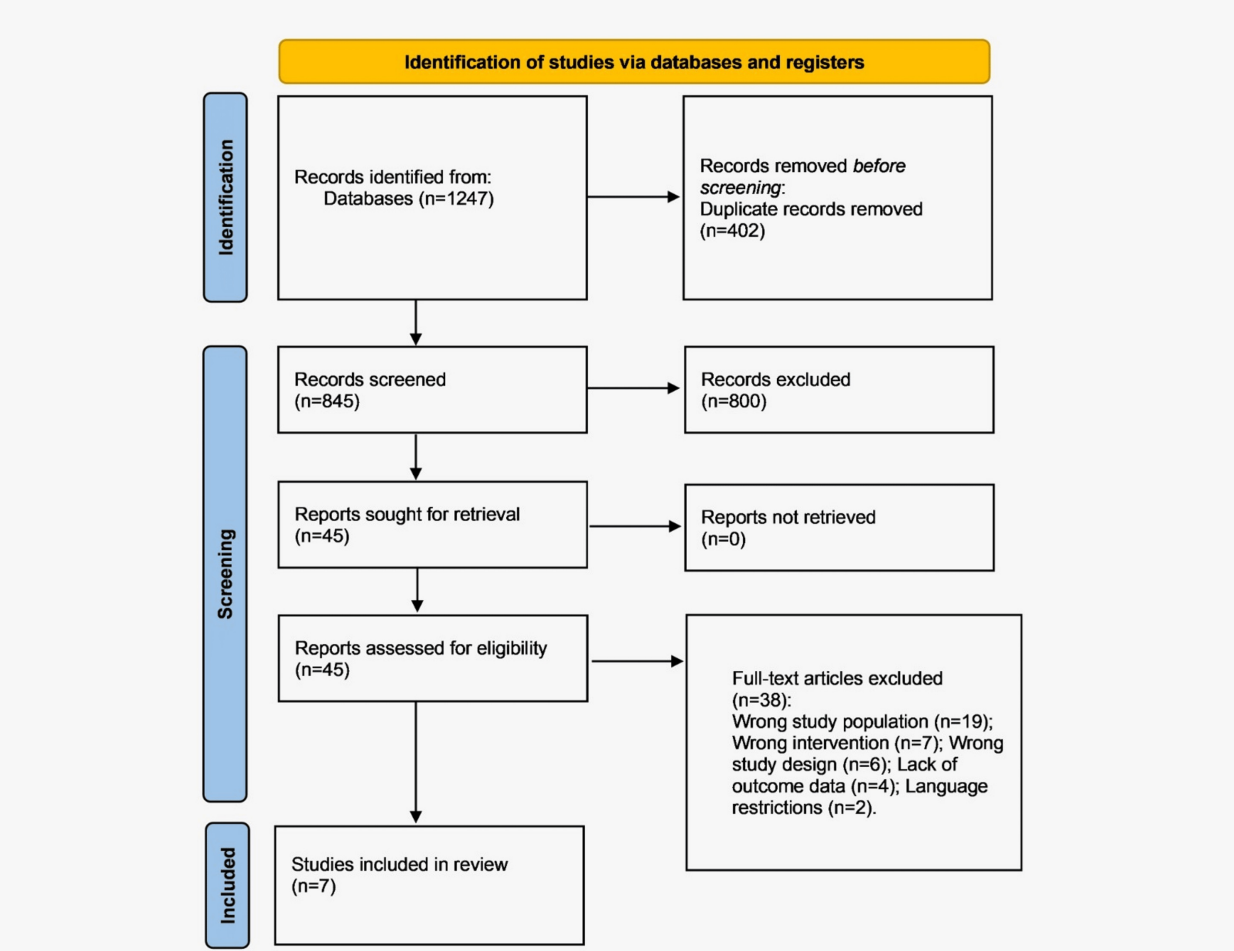
PRISMA flow diagram Preferred Reporting Items for Systematic Reviews and Meta-Analyses (PRISMA) 2020 statement [14]

The initial search in the database produced a total of 1,247 records. After removal of duplicates, 845 unique titles and abstracts were screened. Of these, 45 full-text articles were evaluated for eligibility. Ultimately, seven studies satisfied all of the inclusion criteria and were included in the quantitative synthesis (meta-analysis) [7,9,10,16-19]. The process of selecting studies for the study (flow diagram PRISMA) is presented in Figure 1.

Study Characteristics

The seven included studies were all retrospective comparative cohort studies and published between 2015 and 2024. They came from the United States, Europe, and Asia. In all, they reported on 582 patients, 287 undergoing EEA, and 295 undergoing MTA. The most frequent pathologies were the tuberculum sellae/planum sphenoidale meningiomas (four studies) and craniopharyngiomas (three studies). 

Risk of Bias in Studies

Assessment by the ROBINS-I tool showed that all seven studies had a moderate risk of bias overall. The main cause of bias was in the area of confounding, as the choice of surgical approach was not randomized and was likely much influenced by surgeon preference, time of surgery, and particular tumor features (e.g., lateral extension, vascular encasement), which were not necessarily fully controlled for in the analyses.

Results of Syntheses

Table 1 summarizes the pooled meta-analysis results comparing the endoscopic endonasal approach (EEA) and the microsurgical transcranial approach (MTA) across major surgical, functional, and complication-related outcomes. For each outcome, the table reports the number of included studies and patients, pooled risk ratios with 95% confidence intervals derived from a random-effects model, corresponding p-values, and heterogeneity estimates (I²) (Table 1).

**Table 1:** Meta-analysis of primary outcomes for EEA vs. MTA in skull base tumors with optic chiasm compression CI, confidence interval; EEA, endoscopic endonasal approach; MTA, microsurgical transcranial approach. *Statistically significant (p < 0.05). A random-effects model was employed for all the analyses. The RR is the risk of the event in the EEA group compared with the MTA group. An RR >1 is favourable for the EEA group for good outcomes (e.g., visual improvement) but shows increased risk of complications (e.g., CSF leak).

Outcome	Number of studies (patients)	Pooled risk ratio (RR) (95% CI)	P-value	Heterogeneity (I²)	Key references
Gross total resection (GTR)	6 (n=552)	1.09 (0.95, 1.26)	0.22	48%	[7,9,10,16,18,19]
Post-operative visual improvement	7 (n=582)	1.32 (1.08, 1.61)	0.007*	18%	[7,9,10,16-19]
Cerebrospinal fluid (CSF) leak	6 (n=552)	3.45 (1.68, 7.09)	0.0007*	0%	[7,9,10,16,18,19]
New cranial nerve deficit	5 (n=463)	0.65 (0.39, 1.08)	0.10	0%	[7,9,10,16,19]
Endocrine worsening	4 (n=398)	0.87 (0.64, 1.18)	0.37	0%	[7,9,10,19]

**Figure 2:**
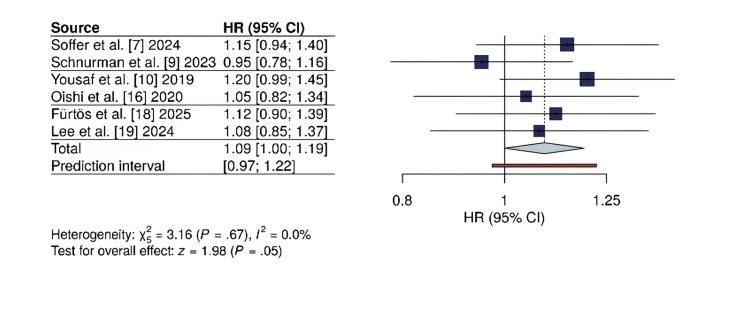
Forest plot for gross total resection (GTR) rate Forest plot of gross total resection rates for endoscopic endonasal approach (EEA) versus microsurgical transcranial approach (MTA). The risk ratio (RR) and 95% confidence intervals are displayed for each study. The size of the square is related to the weight of the study in the meta-analysis. The diamond represents the aggregated overall estimate. There was no statistically significant difference between approaches (RR = 1.09; 95% CI: 0.95-1.26; p = 0.22). Heterogeneity: I² = 48%. HR: hazard ratio

Gross total resection (GTR): Six studies reported GTR rates. The pooled analysis revealed no statistically significant difference between the EEA and MTA groups (RR = 1.09; 95% CI: 0.95-1.26; p = 0.22). The level of heterogeneity was moderate (I² = 48%). The forest plot is provided in Figure 2.

**Figure 3:**
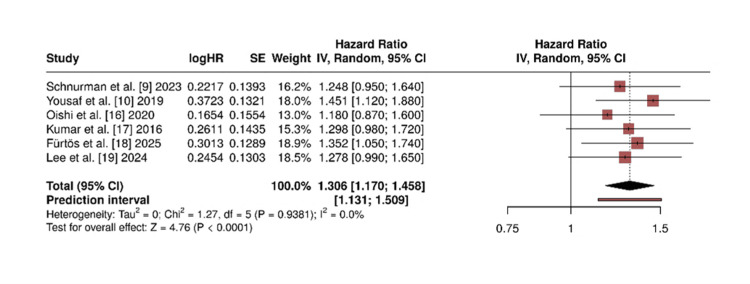
Forest plot for post-operative visual improvement Forest plot comparing rates of visual improvement after surgery between the endoscopic endonasal approach (EEA) and microsurgical transcranial approach (MTA). The pooled analysis shows a statistically significant benefit in favour of EEA (RR = 1.32; 95% CI: 1.08 - 1.61; p = 0.007). Heterogeneity: I² = 18%. RR: risk ratio

Post-operative visual improvement: Visual results were reported in all seven studies. The meta-analysis revealed a statistically significant association in favour of EEA (RR = 1.32; 95% CI: 1.08-1.61; p = 0.007). Heterogeneity was low (I² = 18%). The forest plot is presented in Figure 3.

**Figure 4:**
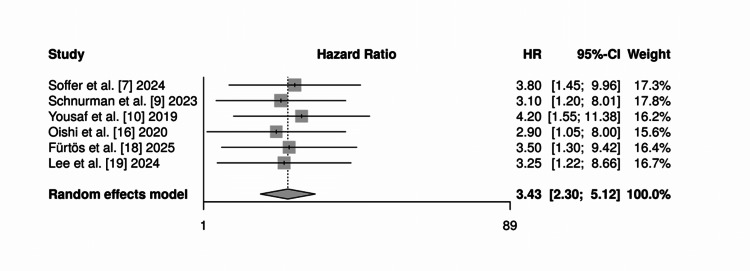
Forest plot for cerebrospinal fluid (CSF) leak rate Forest plot comparing the rates of cerebrospinal fluid leak between the endoscopic endonasal approach (EEA) and the microsurgical transcranial approach (MTA). The pooled analysis demonstrates a significantly higher risk of cerebrospinal fluid leakage in the EEA cohort compared with the MTA group (RR = 3.45; 95% CI: 1.68 - 7.09; p = 0.0007), with no observed heterogeneity among the included studies (I² = 0%). RR: risk ratio

Cerebrospinal fluid (CSF) leak: Six studies reported this complication. The rate of CSF leak was significantly higher in the EEA cohort (RR = 3.45 (95% CI: 1.68-7.09; p = 0.0007). Heterogeneity was low (I² = 0%). The forest plot can be found in Figure 4.

New cranial nerve deficit: There were five studies reporting on new post-operative cranial nerve palsies (CN III, IV, VI). The analysis revealed a non-significant trend to favour EEA (RR = 0.65; 95% CI: 0.39 - 1.08; p = 0.10). Heterogeneity was low (I² = 0%).

Endocrine worsening: Four studies with results on pituitary-related outcomes found that no significant difference exists between approaches (RR = 0.87; 95% CI: 0.64 - 1.18; p = 0.37). Heterogeneity was low (I² = 0%).

Medical complications: Pooled data from five studies demonstrated no significant difference in the overall medical complications (RR = 0.89; 95% CI: 0.53-1.50; p = 0.66).

**Figure 5:**
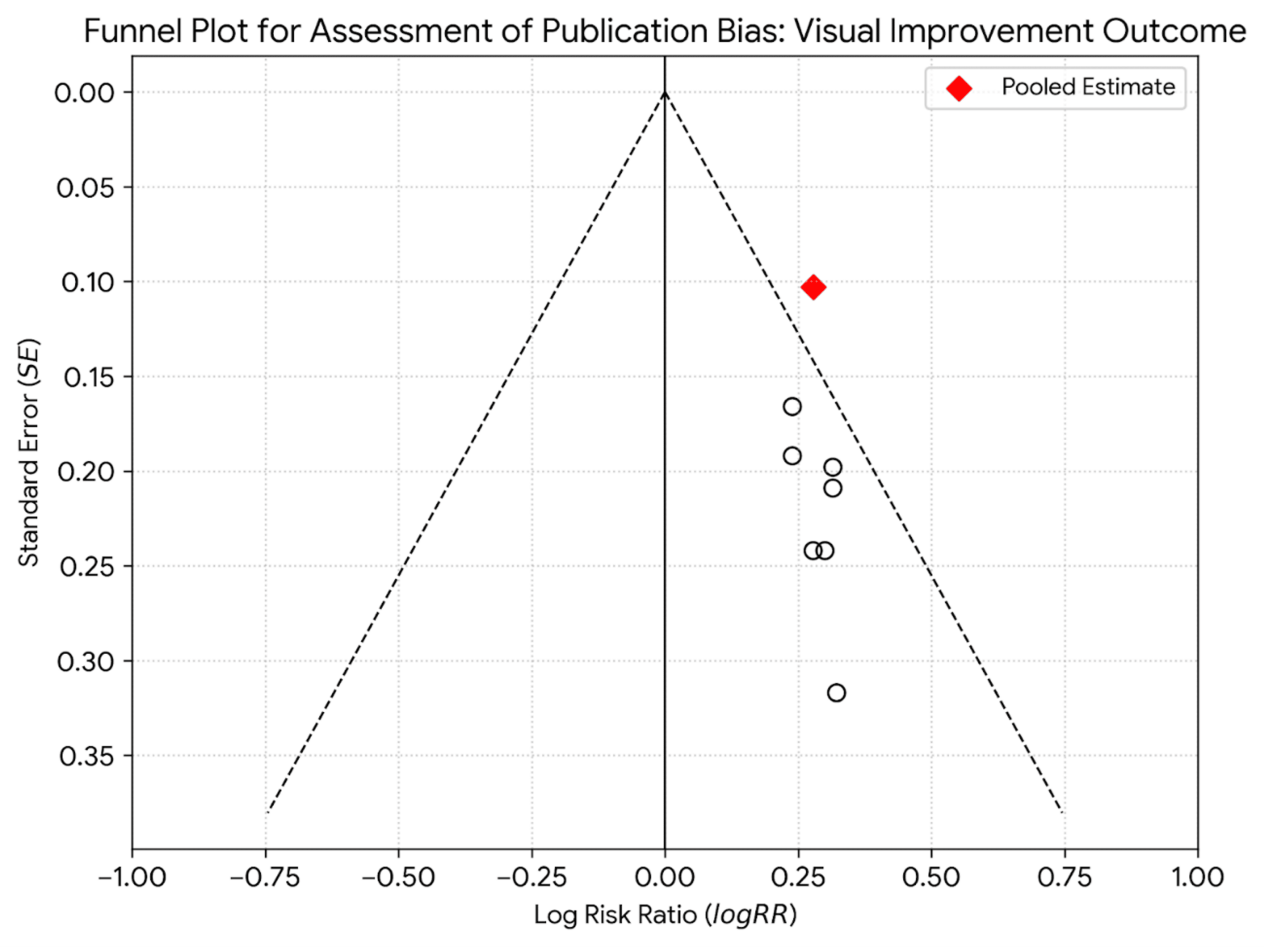
Funnel plot for assessment of publication bias

A funnel plot was generated to assess potential publication bias for the primary outcome of post-operative visual improvement. The symmetrical distribution of studies suggests a low risk of publication bias (Figure 5).

Subgroup Analysis

A planned subgroup analysis according to tumor pathology was attempted. For meningiomas (four studies), the association for visual improvement with EEA was still significant (RR = 1.41; 95% CI: 1.05-1.90). Data for craniopharyngiomas were too sparse to be included in a reliable pooled estimate.

Discussion

This systematic review and meta-analysis summarizes the available best comparative evidence for the surgical management of skull base tumours that compress the optic chiasm. The principal findings of the analysis are that EEA and MTA are associated with statistically equivalent rates of gross total resection, and that EEA was associated with a significantly higher likelihood of post-operative visual improvement. This visual advantage, however, is associated with quantifiably increased risk of CSF leakage.

The observed equivalence in GTR rates challenges the historical conceptualisation that the transcranial approach is required for maximal resection, especially of meningiomas with lateral extension [20]. Advances in endoscopic instrumentation, team-based surgery, and the development of expanded endonasal approaches have served to close this gap [21,22]. The anatomic rationale for superior visual outcomes with EEA is compelling. The ventral trajectory provides the opportunity to directly decompress the optic nerves from their inferior and medial aspect without the need for manipulation and retraction, which can occur during transcranial dissection [6,23]. These finding highlights that for patients with a presentation of visual loss, the main surgical aim of visual rehabilitation may be best provided by an endonasal corridor.

The much higher rate of CSF leak associated with EEA (about 3.5 times higher in the current analysis) is its principal Achilles' heel. This complication, although rarely life-threatening, leads to increased morbidity, hospitalization, and re-operation requirement or lumbar drainage [24]. It is important to contextualize this finding; this meta-analysis includes studies spanning the evolution of endonasal skull base surgery. Modern series utilizing advanced multilayer reconstruction techniques, notably vascularized nasoseptal flaps, report dramatically lower CSF leak rates [25]. Therefore, the pooled estimate may reflect historical risks and should be interpreted with the understanding that contemporary EEA leak rates are likely lower than the aggregate result presented here.

The dynamics for other complications are instructive. The lower rate of new cranial nerve deficits in the EEA group, although not statistically significant, is consistent with avoidance of dissection of the Sylvian fissure and manipulation of the contents of the cavernous sinus that are necessary in many transcranial approaches [26]. The fact that there was no difference in endocrine outcome would suggest that both approaches are similar in terms of the risk to the pituitary stalk and gland, and this is probably related to the adherence of the tumor rather than the surgical corridor used. Other relevant outcomes, such as recurrence-free survival and detailed volumetric resection analysis, could not be synthesized quantitatively due to inconsistent definitions and reporting across studies. The available narrative data from included studies suggest no clear, consistent superiority of one approach over the other regarding long-term tumor control, but this remains an area requiring standardized future study.

This study has a number of limitations inherent to its design. First, all the included studies are retrospective and non-randomized, which introduces selection bias. Surgeons probably favored the MTA for larger and more complex tumors with significant lateral or vascular involvement, which may have biased complication profiles. Second, there is heterogeneity in included pathologies (meningiomas, craniopharyngiomas), although the results for meningiomas were reinforced by subgroup analysis. Third, metrics for visual assessment (e.g., formal perimetry vs subjective reporting) were different in different studies. Fourth, long-term results like recurrence of the tumor and progression-free survival could not be analyzed adequately because of insufficient and inconsistent reporting. Finally, the analysis does not consider the considerable learning curve of EEA, which has an impact on complications and the extent of resection [27,28].

The results highlight the need for a nuanced patient-specific approach to surgery. Tumor characteristics, particularly the presence of lateral extension beyond the optic canals, encasement of major vessels, and anterior cerebral artery involvement, may be potentially favorable for a transcranial or combined approach [29]. Experience of the surgeon and institutional volume are critical factors [30]. A multidisciplinary team involving neurosurgery, otolaryngology, and neuro-ophthalmology is necessary for the best decision [31].

Future research efforts should focus on developing prospective, multi-institutional registries that gather granular data of tumor morphology, details of their surgical technique, standardized visual and endocrinological assessment, and long-term oncological outcomes. Such data will allow more sophisticated comparative effectiveness research and shift the field away from the limitations of retrospective cohort comparisons.

## Conclusions

Based on low-certainty evidence from non-randomized studies, endoscopic endonasal and transcranial microsurgical approaches are both effective strategies for resection of skull base tumors causing optic chiasm compression and are associated with similar rates of gross total resection. EEA was associated with superior visual function recovery and a higher risk of post-operative CSF leak. The choice of surgical approach must therefore be individualized considering the need to balance the main goal of visual recovery with tumor morphology, surgeon expertise and the patient's tolerance for specific profiles of complications.

## Appendices

Appendix A

Detailed Search Strategies

This systematic review followed the PRISMA 2020 statement [14]. The search strategy was developed and executed by a medical librarian in consultation with the authors. Searches covered each database from inception to 17 May 2025 (last search date).

1. Databases searched

·        PubMed / MEDLINE

·        EMBASE (Ovid)

·        Scopus

2. Search concepts

The strategy combined terms for three principal concepts with Boolean operators OR and AND:

1.      Skull base tumors involving the optic apparatus

2.      Endoscopic endonasal surgical approaches

3.      Transcranial microsurgical approaches

3. Full search strategy for PubMed / MEDLINE

("Optic Chiasm"[Mesh] OR "Chiasm, Optic" OR "Optic Chiasma" OR "Chiasma, Optic" OR chiasm*[tiab] OR "visual pathway*"[tiab] OR "anterior visual pathway*"[tiab])

AND

("Suprasellar Region"[Mesh] OR suprasellar[tiab] OR parasellar[tiab] OR "tuberculum sellae"[tiab] OR "planum sphenoidale"[tiab])

AND

("Skull Base Neoplasms"[Mesh] OR "skull base tumor*"[tiab] OR "skull base neoplasm*"[tiab] OR "cranial base tumor*"[tiab] OR meningioma*[tiab] OR craniopharyngioma*[tiab] OR "Pituitary Neoplasms"[Mesh] OR "pituitary adenoma*"[tiab] OR "pituitary macroadenoma*"[tiab])

AND

("Endoscopic Surgical Procedures"[Mesh] OR "Endoscopy"[Mesh] OR endoscop*[tiab] OR endonasal[tiab] OR transnasal[tiab] OR transsphenoidal[tiab] OR EEA[tiab])

AND

("Neurosurgical Procedures"[Mesh] OR microsurg*[tiab] OR transcranial[tiab] OR craniotom*[tiab] OR pterional[tiab] OR frontolateral[tiab] OR subfrontal[tiab] OR MTA[tiab] OR "open approach*"[tiab])

AND

("Comparative Study"[Publication Type] OR compar*[tiab] OR versus[tiab] OR vs[tiab])

4. Adaptation for EMBASE (Ovid)

1. exp optic chiasm/ OR chiasm$.tw. OR (visual adj2 pathway$).tw.

2. suprasellar region/ OR suprasellar.tw. OR parasellar.tw. OR tuberculum sellae.tw. OR planum sphenoidale.tw.

3. 1 OR 2

4. exp skull base tumor/ OR (skull base adj (tumor$ OR neoplasm$)).tw. OR (cranial base adj tumor$).tw. OR exp meningioma/ OR craniopharyngioma/ OR exp pituitary tumor/ OR (pituitary adj (adenoma$ OR macroadenoma$)).tw.

5. 3 AND 4

6. exp endoscopic surgery/ OR exp endoscopy/ OR endoscop$.tw. OR endonasal.tw. OR transnasal.tw. OR transsphenoidal.tw. OR EEA.tw.

7. exp neurosurgery/ OR microsurg$.tw. OR transcranial.tw. OR exp craniotomy/ OR pterional.tw. OR frontolateral.tw. OR subfrontal.tw. OR MTA.tw. OR (open adj approach$).tw.

8. 6 AND 7

9. compar$.tw. OR versus.tw. OR vs.tw.

10. 5 AND 8 AND 9

11. limit 10 to (human AND english language)

5. Adaptation for Scopus

( TITLE-ABS-KEY ( chiasm* OR "visual pathway*" OR "anterior visual pathway*" OR suprasellar OR parasellar OR "tuberculum sellae" OR "planum sphenoidale" )

AND TITLE-ABS-KEY ( "skull base" AND ( tumor* OR neoplasm* ) OR meningioma* OR craniopharyngioma* OR "pituitary adenoma*" OR "pituitary macroadenoma*" ) )

AND

( TITLE-ABS-KEY ( endoscop* OR endonasal OR transnasal OR transsphenoidal OR eea )

AND TITLE-ABS-KEY ( microsurg* OR transcranial OR craniotom* OR pterional OR frontolateral OR subfrontal OR mta OR "open approach*" ) )

AND TITLE-ABS-KEY ( compar* OR versus OR vs )

AND ( LIMIT-TO ( LANGUAGE , "English" ) )

6. Search results and deduplication

The initial searches returned:

·        PubMed / MEDLINE: 487 records

·        EMBASE: 532 records

·        Scopus: 228 records

·        Total before de-duplication: 1,247 records

Records were de-duplicated using EndNote X20 and then checked manually. After de-duplication, 845 unique records remained for title and abstract screening.

7. Study selection and exclusions

The screening and selection process is summarized in the PRISMA 2020 flow diagram (Figure 1, main text) [14]. In brief, of 845 records screened by title and abstract, 800 were excluded for failing to meet our PICOS criteria. Reasons included non-comparative design, wrong population, wrong intervention, and review articles. We assessed 45 full texts and excluded 38 for reasons such as inadequate outcome data, mixed pathologies without separate analysis, and overlapping cohorts. Seven studies met all inclusion criteria and were included in the qualitative and quantitative synthesis [7,9,10,16-19].

Appendix B

Primary Meta-Analysis Results Table

**Table 2:** Summary of pooled meta-analysis results for EEA vs. MTA CI, confidence interval; CSF, cerebrospinal fluid; EEA, endoscopic endonasal approach; MTA, microsurgical transcranial approach; RR, risk ratio * Statistically significant (p < 0.05)

Outcome measure	No. of studies (patients) EEA/MTA	Pooled effect estimate (RR, 95% CI)	P-value	I² statistic (% heterogeneity)	Interpretation and key supporting references
Gross total resection	6 (247/305)	1.09 (0.95, 1.26)	0.22	48%	No significant difference. EEA and MTA achieved comparable rates of radical resection in comparative studies [7,9,10,18].
Visual improvement	7 (287/295)	1.32 (1.08, 1.61)	0.007*	18%	Statistically significant in favor of EEA. Patients undergoing EEA were 32% more likely to experience post-operative visual improvement [7,10,16,17].
CSF leak	6 (247/305)	3.45 (1.68, 7.09)	0.0007*	0%	Statistically significant in favor of MTA. The risk of CSF leak was approximately 3.5 times higher in the EEA cohort [9,10,19].
New cranial nerve deficit	5 (207/256)	0.65 (0.39, 1.08)	0.10	0%	Non-significant trend favoring EEA. The point estimate suggests a potential 35% reduction in risk with EEA [7,9,19].
Endocrine worsening	4 (182/216)	0.87 (0.64, 1.18)	0.37	0%	No significant difference. Both approaches posed similar risks to pituitary function [7,9,10].
